# Supplementary material for: Separable downmodulation of meiotic axis protein deposition and DNA break induction at chromosome ends
Source: eLife. 2026 Oct 16;15:RP110189. doi: 10.7554/eLife.110189 (PMC13675008; doi:10.7554/eLife.110189)
Supplement: Supplementary file 2 [file elife-110189-supp1.docx]

**Supplementary File 1:** SK1 yeast strains utilized in this study.

| **Strain number** | **Genotype** |
| --- | --- |
| AH7797 | *MATa, ho::LYS2, lys2, ura3, leu2::hisG, his3::hisG, trp1::hisG*  *MATalpha, ho::LYS2, lys2, URA3, LEU2, HIS3, TRP1* |
| AH5217 | *MATa, ho::LYS2, lys2, leu2::hisG, trp1::hisG, ura3, ARG4, his3::hisG, his4B::LEU2, dmc1Δ::ARG4*  *MATalpha, ho::LYS2, lys2, leu2::hisG, TRP1, ura3, arg4-Bgl2, his4B::LEU2, dmc1Δ::ARG4* |
| AH3083 | *MATa, ho::LYS2, lys2, leu2::hisG, his4X::LEU2-URA3, ura3, his3::hisG, trp1::hisG, arg4-Bgl2, dmc1Δ::ARG4, dot1Δ::TRP1*  *MATalpha, ho::LYS2, lys2, leu2::hisG, his4B::LEU2, HIS3, trp1::hisG, ura3,arg4-nsp, dmc1Δ::ARG4, dot1Δ::TRP1* |
| AH2908 | *MATa, ho::LYS2, lys2, leu2::hisG, his4B::LEU2, ura3, arg4-Bgl2, dmc1 Δ::ARG4, sir3::HIS3*  *MATalpha, ho::LYS2, lys2, leu2::hisG, his4B::LEU2, ura3, arg4-Bgl2, dmc1Δ::ARG4, sir3::HIS3* |
| AH13037 | *MATalpha, ho::LYS2, leu2::hisG, ura3, his4B::LEU2, trp1::hisG, dot1Δ::TRP1, arg4-Bgl2, dmc1Δ::ARG4, sir3::HIS3*  *MATa, ho::LYS2, leu2::hisG, ura3, trp1::hisG, dot1Δ::TRP1, arg4-Bgl2, dmc1Δ::ARG4, sir3::HIS3* |
| AH8967 | *MATa, ho::LYS2, lys2, ura3, LEU2, his3::hisG, trp1::hisG,*  *sir3::HIS3, ndt80Δ::TRP1*  *MATalpha, ho::LYS2, lys2, ura3::YIplac211::URA3, LEU2, his3::hisG, trp1::hisG, sir3::HIS3, ndt80Δ::TRP1* |
| AH6179 | *MATa, ho::LYS2, lys2, HIS, URA, LEU, TRP, ndt80Δ::TRP1*  *MATalpha, ho::LYS2, lys2, leu2::hisG, his4X, ura3, trp1::hisG, ndt80Δ::TRP1* |
| AH13257 | *MATa, ho::LYS2, leu2::hisG, trp1::hisG, dot1Δ::TRP1, sir3::HIS3*  *MATalpha, ho::LYS2, leu2::hisG, trp1::hisG, dot1Δ::TRP1, sir3::HIS3* |
| AH2861 | *MATalpha, ho::LYS2, lys2, ura3, leu2::hisG, his3::hisG, trp1::hisG, sir3::HIS3*  *MATa, ho::LYS2, lys2, ura3, leu2::hisG, his3::hisG, trp1::hisG, sir3::HIS3* |
| AH8104 | *MATa, lys2, ho::LYS2, trp1::hisG, his3::hisG, LEU2, URA3, dot1Δ::TRP1*  *MATalpha, lys2, ho::LYS2, trp1::hisG, HIS3, leu2::hisG, ura3, dot1Δ::TRP1* |
| AH13299 | *MATalpha, ho::LYS2, lys2, ura3, leu2::hisG, his4X-LEU2(BamHI)-URA3, arg4-nsp, hht1-K79R, hht2-K79R*  *MATa, ho::LYS2, lys2, ura3, leu2::hisG, his4B-LEU2(MluI), arg4-bgl*  *hht1-K79R, hht2-K79R* |
