# Supplementary material for: Separable downmodulation of meiotic axis protein deposition and DNA break induction at chromosome ends
Source: eLife. 2026 Oct 16;15:RP110189. doi: 10.7554/eLife.110189 (PMC13675008; doi:10.7554/eLife.110189)
Supplement: Supplementary file 3 [file elife-110189-supp2.docx]

**Supplementary File 2:** ChIP-seq datasets utilized in this study.

| **Sample** | **Time** | **Antibody** | **Type** | **GEO accession numbers** | **References** |
| --- | --- | --- | --- | --- | --- |
| Wild type | 3 hr in meiosis | Anti-Red1 | ChIP-seq | GSE69232, GSE115092 | ^1,2^ |
| Wild type | 3 hr in meiosis | Anti-Hop1 | ChIP-seq | GSE105111 | ^3^ |
| Rec8::3HA | 3 hr in meiosis | Anti-HA | ChIP-seq | GSE69232 | ^1^ |
| SK1 WT / S288c | 3 hr in meiosis | Anti-Red1 | ChIP-seq | GSE114731 | ^4^ |
| S288c chrIV-chrI, *cen1Δ* /SK1 chrIV-chrI, *cen1Δ* | 3 hr in meiosis | Anti-Red1 | ChIP-seq | GSE114731 | ^4^ |
| S288c chrIV-chrI, *cen4Δ*/SK1 chrIV-chrI, *cen4Δ* | 3 hr in meiosis | Anti-Red1 | ChIP-seq | GSE114731 | ^4^ |
| *rec8Δ* | 3 hr in meiosis | Anti-Red1 | ChIP-seq + SNPChIP-seq | GSE156040 | ^5^ |
| *hop1-phd* | 3 hr in meiosis | Anti-Red1 | ChIP-seq + SNPChIP-seq | GSE156040 | ^5^ |
| *hop1-phd rec8Δ* | 3 hr in meiosis | Anti-Red1 | ChIP-seq + SNPChIP-seq | GSE156040 | ^5^ |
| Wild type | 4 hr in meiosis | Anti- H4K44ac | ChIP-seq | GSE59005 | ^6^ |
| Wild type | 4 hr in meiosis | Anti-H3K56ac | ChIP-seq | GSE59005 | ^6^ |
| Wild type | 4 hr in meiosis | Anti-H4 | ChIP-seq | GSE59005 | ^6^ |
| Wild type | 4 hr in meiosis | Anti-H3 | ChIP-seq | GSE59005 | ^6^ |
| Wild type | 4 hr in meiosis | Anti-H3K4me3 | ChIP-seq | GSE67907 | ^7^ |
| Wild type | 3 hr in meiosis | Anti-H3K79me3 | ChIP-seq | GSE287129 | This study |
| *dot1Δ* | 3 hr in meiosis | Anti-Red1 | ChIP-seq + SNPChIP-seq | GSE115092 | ^1,2^ |
| *set1Δ* | 3 hr in meiosis | Anti-Red1 | ChIP-seq + SNPChIP-seq | GSE115092 | ^1,2^ |
| Wild type | Mid-log phase | Anti-Sir3 | ChIP-seq | GSE287129 | This study |
| Wild type | 0 hr in meiosis (post-induction) | Anti-Sir3 | ChIP-seq | GSE287129 | This study |
| Wild type | 3 hr in meiosis | Anti-Sir3 | ChIP-seq | GSE287129 | This study |
| *sir3* | 3 hr in meiosis | Anti-Red1 | ChIP-seq + SNPChIP-seq | GSE287129 | This study |
| *sir3 dot1Δ* | 3 hr in meiosis | Anti-Red1 | ChIP-seq + SNPChIP-seq | GSE287129 | This study |
| *hht1-K79R, hht2-K79R* | 3 hr in meiosis | Anti-Red1 | ChIP-seq + SNPChIP-seq | GSE287129 | This study |

**References**

1	Sun, X. *et al.* Transcription dynamically patterns the meiotic chromosome-axis interface. *eLife* **4**, 1–23 (2015). <https://doi.org/10.7554/eLife.07424>

2	Vale-Silva, L. A., Markowitz, T. E. & Hochwagen, A. SNP-ChIP: A versatile and tag-free method to quantify changes in protein binding across the genome. *BMC Genomics* **20**, 1–10 (2019). <https://doi.org/10.1186/s12864-018-5368-4>

3	Subramanian, V. V. *et al.* Persistent DNA-break potential near telomeres increases initiation of meiotic recombination on short chromosomes. *Nature Communications* **10**, 1–15 (2019). <https://doi.org/10.1038/s41467-019-08875-x>

4	Luo, J. *et al.* Synthetic chromosome fusion: Effects on mitotic and meiotic genome structure and function. *Cell Genomics* **3** (2023). <https://doi.org/10.1016/j.xgen.2023.100439>

5	Heldrich, J. *et al.* Two pathways drive meiotic chromosome axis assembly in *Saccharomyces cerevisiae*. *Nucleic Acids Research* **50**, 4545–4556 (2022).

6	Hu, J. *et al.* H4K44 Acetylation Facilitates Chromatin Accessibility during Meiosis. *Cell Reports* **13**, 1772–1780 (2015). <https://doi.org/10.1016/j.celrep.2015.10.070>

7	Zhu, X. & Keeney, S. High-resolution global analysis of the influences of Bas1 and Ino4 transcription factors on meiotic DNA break distributions in *Saccharomyces cerevisiae*. *Genetics* **201**, 525–542 (2015). <https://doi.org/10.1534/genetics.115.178293>
