# Supplementary material for: Separable downmodulation of meiotic axis protein deposition and DNA break induction at chromosome ends
Source: eLife. 2026 Oct 16;15:RP110189. doi: 10.7554/eLife.110189 (PMC13675008; doi:10.7554/eLife.110189)
Supplement: Supplementary file 4 [file elife-110189-supp3.docx]

**Supplementary File 3:** Spo11-Oligos, RNA-seq, TrAEL-seq and MNase-seq datasets utilized in this study.

| **Sample** | **Time point** | **Methodology** | **GEO accession numbers** | **Reference** |
| --- | --- | --- | --- | --- |
| Wild type | 4 hr in meiosis | Spo11-Oligos | GSE48299 | ^1^ |
| *sir2Δ* | 4 hr in meiosis | Spo11-Oligos | GSE122882 | ^2^ |
| *ndt80Δ* | 3 hr in meiosis | RNA-seq | GSE287127 | This study |
| *sir3 ndt80Δ* | 3 hr in meiosis | RNA-seq | GSE287127 | This study |
| *dmc1Δ* | 5 hr in meiosis | TrAEL-seq | GSE287130 | This study |
| *sir3 dmc1Δ* | 5 hr in meiosis | TrAEL-seq | GSE287130 | This study |
| *dot1Δ dmc1Δ* | 5 hr in meiosis | TrAEL-seq | GSE287130 | This study |
| *sir3 dot1Δ dmc1Δ* | 5 hr in meiosis | TrAEL-seq | GSE287130 | This study |
| Wild type | 3 hr in meiosis | MNase-seq | GSE307629 | This study |
| *sir3* | 3 hr in meiosis | MNase-seq | GSE307629 | This study |
| *dot1Δ* | 3 hr in meiosis | MNase-seq | GSE307629 | This study |
| *sir3 dot1Δ* | 3 hr in meiosis | MNase-seq | GSE307629 | This study |

**References**

1	Thacker, D., Mohibullah, N., Zhu, X. & Keeney, S. Homologue engagement controls meiotic DNA break number and distribution. *Nature* **510**, 241–246 (2014). <https://doi.org/10.1038/nature13120>

2	Subramanian, V. V. *et al.* Persistent DNA-break potential near telomeres increases initiation of meiotic recombination on short chromosomes. *Nature Communications* **10**, 1–15 (2019). <https://doi.org/10.1038/s41467-019-08875-x>
