# Supplementary material for: Separable downmodulation of meiotic axis protein deposition and DNA break induction at chromosome ends
Source: eLife. 2026 Oct 16;15:RP110189. doi: 10.7554/eLife.110189 (PMC13675008; doi:10.7554/eLife.110189)
Supplement: Peer review history

# Editor report: eLife Assessment

**DOI:** 10.7554/eLife.110189.4.sa0

**Authors:** Akira Shinohara

**Affiliations:** The University of Osaka Japan

**Keywords:** Solid, Valuable

This **valuable** paper describes the regulation of the association of meiotic chromosome axis proteins on chromosome ends with sub-telomeric elements in budding yeast. The genome-wide analyses of binding of chromosome components as well as chromatin regulators, complemented with the mapping of meiotic DNA double-strand breaks on chromosome ends, provided **solid** evidence to support the authors' conclusion. The results in the paper are of interest to researchers studying meiotic recombination and the structure of genomes and chromosomes.

# Referee report: Reviewer #1 (Public review)

**DOI:** 10.7554/eLife.110189.4.sa1

[Editors' note: this version has been assessed by the Reviewing Editor without further input from the original reviewers. The authors have addressed the comments raised in the previous round of review.]

Meiotic recombination at chromosome ends can be deleterious, and its initiation-the programmed formation of DSBs-has long been known to be suppressed. However, the underlying mechanisms of this suppression remained unclear. A bottleneck has been the repetitive sequences embedded within chromosome ends, which make them challenging to analyze using genomic approaches. The authors addressed this issue by developing a new computational pipeline that reliably maps ChIP-seq reads and other genomic data, enabling exploration of previously inaccessible yet biologically important regions of the genome.

In budding yeast, chromosome ends (~20 kb) show depletion of axis proteins (Red1 and Hop1) important for recruiting DSB-forming proteins. Using their newly developed pipeline, the authors reanalyzed previously published datasets and data generated in this study, revealing here-to-fore-unseen details at chromosome ends. While axis proteins are depleted at chromosome ends, the meiotic cohesin component Rec8 is not. Y' elements play a crucial role in this suppression. The suppression does not depend on the physical chromosome ends but on cis-acting elements. Dot1 suppresses Red1 recruitment at chromosome ends but promotes it in interior regions. Sir complex renders subtelomeric chromatin inaccessible to the DSB-forming machinery.

The high-quality data and extensive analyses provide important insights into the mechanisms that suppress meiotic DSB formation at chromosome ends.

Comments on latest version:

I have checked the authors' responses and the revised analyses. I think they have adequately addressed my main concerns, particularly regarding the quantitative analyses of the chromosome fusion and SK1/S288c comparisons. I have no further comments and am content for you to proceed.

# Referee report: Reviewer #2 (Public review)

**DOI:** 10.7554/eLife.110189.4.sa2

Summary:

In this manuscript, Raghavan and his colleagues sought to identify cis-acting elements and/or protein factors that limit meiotic crossover at chromosome ends. This limitation is important for avoiding chromosome rearrangements and preventing chromosome mis-segregation.

By comparing protein axis recruitment in SK1 and S288C background, which differ in their number and distribution of Y' elements, the authors show that Y' element have a limited impact on axis protein enrichment. Genetic analyses coupled with ChIP experiments revealed that the differential binding of the Red1 protein in subtelomeric regions requires the methyltransferase Dot1. Interestingly, the lack of Red1 depletion in subtelomeric regions in this mutant does not impact DSB formation. Another surprising finding is that deleting DOT1 has no effect on Red1 loading in the absence of the silencing factor Sir3. Unlike Dot1, Sir3 directly impacts DSB formation, probably by limiting promoter access to Spo11. As now clearly stated in the abstract and the discussion, this explains only a small part of the low levels of DSBs forming in subtelomeric regions and the main mechanisms suppressing crossover close to the ends of chromosomes remain to be deciphered.

Strengths:

This work provides intriguing observations, such as the impact of Dot1 and Sir3 on Red1 loading and the uncoupling of Red1 loading and DSB induction in subtelomeric regions.

The separation of axis protein deposition and DSB induction observed in the absence of Dot1 is interesting because it rules out the possibility that the binding pattern of these proteins is sufficient to explain the low level of DSB in subtelomeric regions.

The demonstration that Sir3 suppresses the induction of DSBs by limiting the openness of promoters in subtelomeric regions is convincing.

Weaknesses:

Sir3's impact on DSB induction is compelling, yet it only accounts for a small proportion of DSB depletion in subtelomeric regions. Thus, the main mechanisms suppressing crossover close to the ends of chromosomes remain to be deciphered. [Update: these limitations have been added to the text.]

# Author comment: Author response

**DOI:** 10.7554/eLife.110189.4.sa3

**Authors:** Adhithi R Raghavan, Kieron May, Vijayalakshmi V Subramanian, Hannah G Blitzblau, Neem J Patel, Jonathan Houseley, Andreas Hochwagen

**Affiliations:** New York University New York United States; Babraham Institute Cambridge United Kingdom; Indian Institute of Science Education and Research Tirupati Tirupati India; New York University New York United States; New York University New York United States; The Babraham Institute Cambridge United Kingdom; New York University New York United States

The following is the authors’ response to the previous reviews

**Public Reviews:**

**Reviewer #1 (Public review):**

The revised manuscript includes several useful additions, and I appreciate the efforts to clarify parts of the analysis. The dataset remains valuable. However, several key issues raised previously are not yet fully resolved and continue to limit the clarity of the main conclusions.

(1) I appreciate that the authors guide the reader to the relevant regions in the analysis of chromosome fusions (Fig. 2b). However, these subtelomeric regions are not clearly visualized, making it difficult to compare fused and unfused profiles, even though the conclusions rely largely on visual inspection of them. A more direct comparison between fused and unfused ends, together with quantitative summaries (e.g., binned Red1 enrichment and comparisons with internal regions), would make this experiment more convincing.

Thank you for this suggestion. Figure 2 – figure supplement 1 now shows Red1 enrichment in 20-kb bins tiling in from the fusion points to more clearly show that there is no significant difference in Red1 enrichment between fused and unfused chromosomes. These data are consistent with the model that Red1 enrichment is not affected by the presence of telomeres and imply that Red1 under-enrichment near telomeres is primarily encoded in cis.

(2) The SK1/S288c comparison (Fig. 2c) is an excellent approach, but is currently presented just as profiles, which again requires substantial effort from the reader to extract the relevant information. A systematic analysis across all informative chromosome ends-for example, comparing Red1 levels in syntenic regions using binned log2 fold-change-would more directly test the proposed in cis effect (L168) and clarify the contribution and range of Y'-associated effects. Other factors (e.g. distance from chromosome ends) could also be assessed within this framework.

Thank you. Figure 2 – figure supplement 2 now shows the profiles placed in register using peak distribution. This analysis demonstrates that registered S288c and SK1 profiles have the same enrichment of Red1, indicating that there are no detectable long-range effects of Y’ elements or other telomere-associated sequences on the neighboring axis binding sites. Figure 2 – figure supplement 3a further quantifies the effect of registering profiles and separates the data based whether Y’ elements are present. These data are consistent with the interpretation that the presence of Y’ elements primarily affects the average axis protein enrichment profiles by displacing strong axis protein binding sites towards the chromosome interior. Our analyses also indicate that this effect is not limited to the Y’ elements as other telomere-associated sequences have a very similar effect on axis protein distribution near chromosome ends.

Related to this, it is unclear if Y' elements themselves exhibit lower Red1 binding than the genome average. Providing the mean Red1 signal per Y' element would clarify this point and may also aid interpretation of the relationship between coding density and Red1 enrichment.

Figure 1 – figure supplement 4a and Figure 2 – figure supplement 3b now show that the mean Red1 enrichment on Y’ elements is on average lower than in the rest of the genome. However, as shown in Figure 2 – figure supplement 3b, this effect is not unique to Y’ elements as other telomere-associated sequences show a very similar level of depletion.

(3) The Dot1-Sir3 section is now simpler. However, I still find it difficult to follow the underlying rationale. In particular, it is unclear why a Dot1 function dependent on H3K79 methylation is introduced, given that the data in the previous section suggest H3K79 methylation is dispensable for subtelomeric Red1 depletion. A clearer statement of the authors' working model would be helpful.

We apologize for this confusion. We restructured this section in an attempt to clarify the link between Dot1 activity and Sir3.

**Reviewer #2 (Public review):**

Summary:

In this manuscript, Raghavan and his colleagues sought to identify cis-acting elements and/or protein factors that limit meiotic crossover at chromosome ends. This limitation is important for avoiding chromosome rearrangements and preventing chromosome mis-segregation.

By comparing protein axis recruitment in SK1 and S288C background, which differ in their number and distribution of Y' elements, the authors show that Y' element have a limited impact on axis protein enrichment. Genetic analyses coupled with ChIP experiments revealed that the differential binding of the Red1 protein in subtelomeric regions requires the methyltransferase Dot1. Interestingly, the lack of Red1 depletion in subtelomeric regions in this mutant does not impact DSB formation. Another surprising finding is that deleting DOT1 has no effect on Red1 loading in the absence of the silencing factor Sir3. Unlike Dot1, Sir3 directly impacts DSB formation, probably by limiting promoter access to Spo11. As now clearly stated in the abstract and the discussion, this explains only a small part of the low levels of DSBs forming in subtelomeric regions and the main mechanisms suppressing crossover close to the ends of chromosomes remain to be deciphered.

Strengths:

This work provides intriguing observations, such as the impact of Dot1 and Sir3 on Red1 loading and the uncoupling of Red1 loading and DSB induction in subtelomeric regions.

The separation of axis protein deposition and DSB induction observed in the absence of Dot1 is interesting because it rules out the possibility that the binding pattern of these proteins is sufficient to explain the low level of DSB in subtelomeric regions.

The demonstration that Sir3 suppresses the induction of DSBs by limiting the openness of promoters in subtelomeric regions is convincing.

Weaknesses:

The section examining the impact of Dot1 and Sir3 remains complex, which is partly inherent to the intricate relationship between Dot1 and Sir3. However, the authors conclude that Dot1 acts independently of its catalytic activity based on the phenotype of the H3K79R mutant phenotype. Although this is possible it is not fully demonstrated as the H3K79R mutant may exhibit its own phenotype independently of Dot1. Unless the authors test the impact of the catalytic dead mutant Dot1-G401R on axis protein enrichment at subtelomeres they cannot claim that Dot1 act independently of its catalytic activity.

Thank you. We softened the relevant statements and do not invoke Dot1 catalytic activity.

Sir3's impact on DSB induction is compelling, yet it only accounts for a small proportion of DSB depletion in subtelomeric regions. Thus, the main mechanisms suppressing crossover close to the ends of chromosomes remain to be deciphered.

We explicitly state the fact that further regulation remains to be discovered in the abstract, results, and discussion.
